# CT-based and manual external skull measurements for Chiari-like malformation and syringomyelia in Pomeranians

**DOI:** 10.1371/journal.pone.0313203

**Published:** 2024-11-25

**Authors:** Koen M. Santifort, Sophie Bellekom, Ines Carrera, Paul J. J. Mandigers

**Affiliations:** 1 IVC Evidensia Referral Hospital Arnhem, Arnhem, Netherlands; 2 IVC Evidensia Referral Hospital Hart van Brabant, Waalwijk, Netherlands; 3 Expertise Centre of Genetics, Department of Clinical Sciences, Faculty of Veterinary Medicine, Utrecht University, Utrecht, Netherlands; 4 Vet Oracle Teleradiology, Norfolk, United Kingdom; Wrocław University of Environmental and Life Sciences: Uniwersytet Przyrodniczy we Wroclawiu, POLAND

## Abstract

Studies in Pomeranians as well as other breeds have looked at clinical signs or external features as predictors of Chiari-like malformation (CM) and syringomyelia (SM). The aim of this study is to describe and analyze morphometric factors of the skull of Pomeranians with and without CM/SM by means of CT as well as manual external measurements. Ninety-two Pomeranians >12 months of age were included that underwent both CT and MRI studies of the head and cervicothoracic vertebral column. Two observers independently reviewed the CT imaging studies and performed quantitative measurements. External measurements were taken from the head of dogs when under general anesthesia using a tape measure and a caliper. Externally measured mandible length was associated with the probability of having SM (p = 0.043). Mandible length was moderately correlated with weight (Pearson correlation coefficient = 0.585, p < 0.001). A cutoff for mandible length of 58 mm yielded a sensitivity of 96% (95% confidence interval 89–100%), meaning dogs in this study population with a mandible length shorter than 58 mm were highly likely to have SM. The measurement of the length of the mandible could help to determine the probability of a Pomeranian having SM, especially when combined with the presence or absence of owner reported clinicals signs as shown in previous studies.

## Introduction

Chiari-like malformation (CM) and syringomyelia (SM) are disorders that can have devastating effects on the quality of life of dogs. The pathophysiology and diagnostic imaging characteristics of dogs with Chiari-like malformation (CM) with or without syringomyelia (SM) have been subject of considerable interest over the last two decades [1–4]. Morphometric as well as clinical studies in, among other breeds, Cavalier King Charles Spaniels (CKCS), Griffon Bruxellois, and Chihuahuas have provided veterinarians and owners with valuable information on which clinical decision-making, future research and breeding programs can be based [5–18].

Recent studies in the CKCS and Pomeranian dog breeds have linked specific clinical (owner-reported) signs to CM/SM status [19–22]. Findings of these studies are of direct clinical value. Evaluation of clinical signs can help estimate the likelihood of a particular dog having CM/SM or not. Thus, clinicians can identify those dogs that are more likely to have CM/SM by recognizing the clinical signs associated with CM/SM in dogs. The formal diagnosis of CM/SM still requires magnetic resonance imaging (MRI).

The results of morphometric studies in breeds other than the Pomeranian have contributed to the understanding of the pathogenesis of CM/SM in dogs [10–16]. Along with the evaluation of clinical signs, morphometric features may be used to assess the risk of SM in dogs of those breeds. Recently, we have published the first large morphometric study focused on Pomeranians with and without CM/SM [23, 24]. As that study focused MRI and CT of the craniocervical region, application of these results clinically would still require sedation (CT) or anesthesia (MRI). Currently, morphometrical assessments not necessitating sedation or anesthesia have not been studied in relation to CM/SM in Pomeranians.

With this study, we aim to:

1. Describe and analyze morphometric factors of the skull of Pomeranians with and without CM/SM by means of CT as well as manual external measurements.
2. Analyze for differences between CM/SM classification groups for CT measurements and analyze for associations of CM/SM status with manual external measurements (i.e. if manual external measurements can help to assess the risk of CM/SM).
3. Report consistency and correlation between CT-based measurements and manual external measurements.

## Materials and methods

For this prospective study, Pomeranians underwent both CT and MRI studies of the head and cervicothoracic vertebral column at one institution (IVC Evidensia Small Animal Hospital Arnhem) during the period of February 2022 to October 2023. These dogs were presented to the neurology department by their owners for a so-called ‘screening MRI and CT scan’ specifically to diagnose or exclude CM/SM (MRI) and acquire CT images for scientific research. Dogs with a prior history or diagnosis of CNS disease on MRI were excluded with exception of dogs affected by the following disorders: CM/SM, ventriculomegaly, supracollicular fluid accumulation, findings related to craniocervical junction abnormalities (CJA), or non-structural disorders such as epilepsy or paroxysmal dyskinesia. Dogs with MRI or CT studies with artifacts or insufficient image quality that did not allow for accurate assessments or measurements were excluded, as were dogs aged <12 months (skeletally (skull) immature dogs). Informed owner consent was obtained.

MRI and CT studies were performed under general anesthesia (individualized anesthetic protocols) with a high-field MRI scanner (1.5T Canon Vantage Elan, The Netherlands) and 16-slice CT scanner (Siemens SOMATOM.go, The Netherlands). Dogs were positioned in sternal recumbency on the horizontal surface of the table with the head in a flexible coil (MRI) or a head rest (CT), both resulting in elevation of the head of about 2–3 cm above the table. The scanned region included the head and cervicothoracic vertebral column up to at least the 4th thoracic vertebra. CT scans were performed with the following parameters: 130 kVp tube voltage, 80 and 220 mAs tube current, 256 × 256 image matrix, 0.6 and 0.8 mm slice thickness, 0.4 and 0.6 mm slice increment, 1.0 s rotation time, and a pitch of 0.6. A bone algorithm was used for image reconstruction in transverse, dorsal, and sagittal planes.

The animal study protocol was reviewed and ethically approved by the Animal Welfare Body Utrecht (Utrecht University, The Netherlands). The studies were conducted in accordance with the local legislation and institutional requirements.

### CT measurements

**Fig 1:**
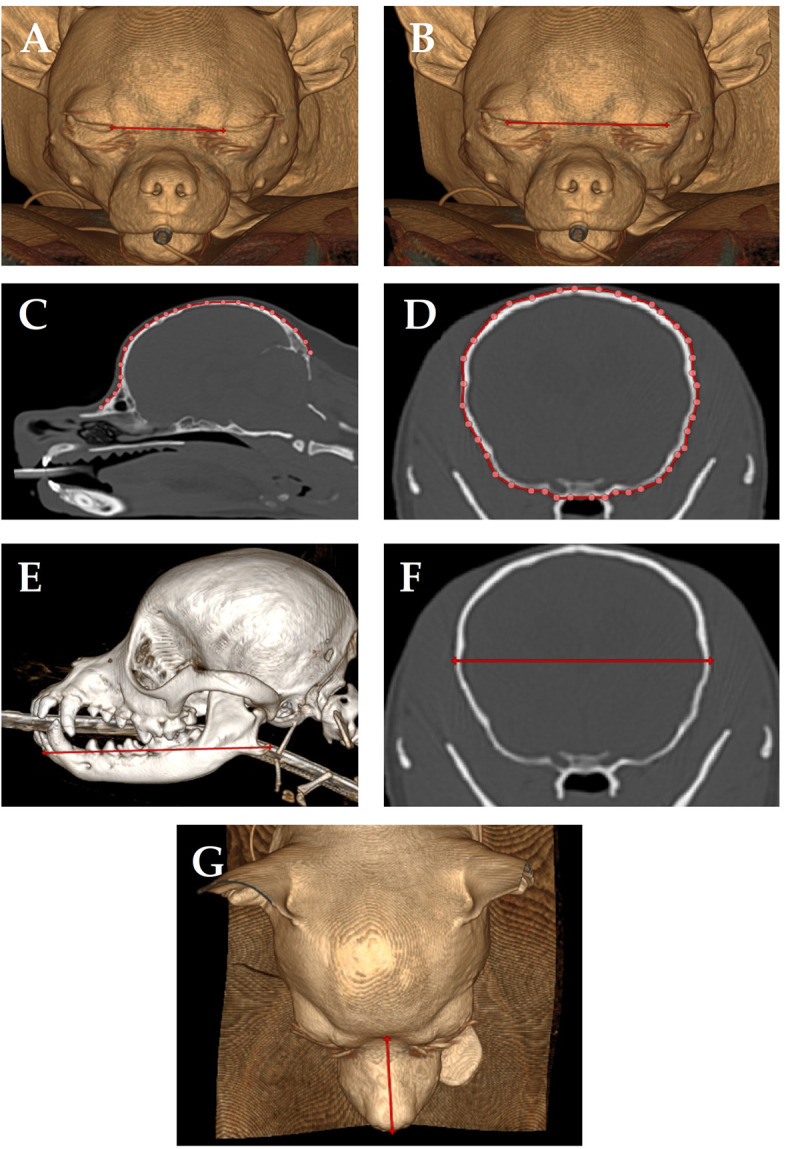
CT-based measurements. (a) distance between medial canthi of the eyes (intercanthal distance, 3D reconstruction); (b) distance between the center of both eyes (interpupillary distance, 3D reconstruction); (c) distance from the stop to the external occipital protuberance following the skull borders (CT-based cranial length) (sagittal reconstruction); (d) circumference of the skull at the level of the orbital fissure (transverse); (e) length of the mandible (rostral tip of the mandible to the angular process) (3D reconstruction (bone)); (f) maximum width of the skull at the level of the orbital fissure; (g) length of the dorsal aspect of the nose from the planum to the stop (muzzle length; 3D reconstruction).

Two observers (KS (Diplomate European College of Veterinary Neurology) and SB (resident European College of Veterinary Diagnostic Imaging)) independently reviewed the CT imaging studies and performed quantitative measurements using imaging software (RadiAnt DICOM Viewer [Software version 2023.1]). One observer (KS) performed all of the measurements twice. CT measurements were performed using manual tracing tools. Measurements included (Fig 1):

- Distance between medial canthi of the eyes (intercanthal distance, 3D reconstruction) (Fig 1A);
- Distance between the center of both eyes (interpupillary distance, 3D reconstruction) (Fig 1B);
- Distance from the stop to the external occipital protuberance following the skull borders (CT-based cranial length) (sagittal reconstruction) (Fig 1C);
- Circumference of the skull at the level of the orbital fissure (transverse) (Fig 1D);
- Length of the mandible (rostral tip of the mandible to the angular process) (3D reconstruction (bone)) (Fig 1E);
- Maximum width of the skull at the level of the orbital fissure (Fig 1F), and;
- Length of the dorsal aspect of the nose from the planum to the stop (muzzle length; 3D reconstruction) (Fig 1G).

CM/SM classifications based on MRI scans had already been performed in a previous study [21, 22]. CM and SM status was classified as CM normal = CM0 and CM abnormal = CM1 and CM2, and SM normal = SM0 and SM abnormal = SM1 and SM2. These classifications were described previously [21, 23, 24] and are briefly defined as: CM0 = no cerebellar herniation or impaction, CM1 = cerebellar impaction, CM2 = cerebellar herniation and SM0 = no syringomyelia, SM1 = symmetrical syringomyelia, SM2 = asymmetrical syringomyelia). The means of the measurements performed by the observers were used for final analysis.

Ratios were calculated for the following measurements:

- Interpupillary distance: Intercanthal distance (Ratio 1)
- Circumference of the skull at the level of the orbital fissure: Distance from the stop to the external occipital protuberance following the skull borders (modified cephalic index 1) (Ratio 2)
- Maximum width of the skull at the level of the orbital fissure: Distance from the stop to the external occipital protuberance following the skull borders (modified cephalic index 2) (Ratio 3)
- Ratio 4 = Length of the dorsal aspect of the nose from the planum to the stop (muzzle length): Distance from the stop to the external occipital protuberance following the skull borders (CT-based craniofacial ratio, Ratio 4)
- Length of the dorsal aspect of the nose from the planum to the stop (muzzle length): Length of the mandible (Ratio 5)

### External manual measurements

**Fig 2:**
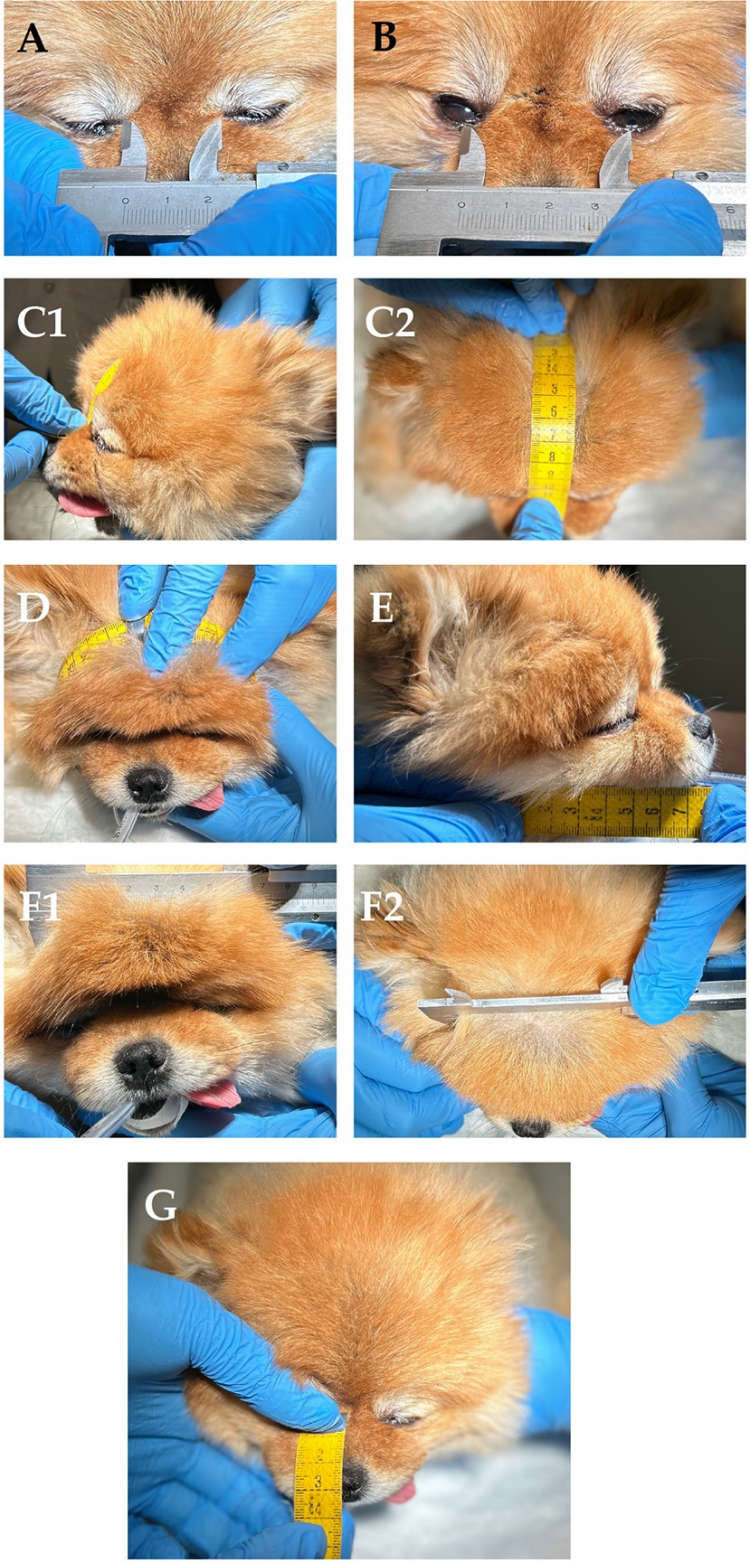
Manual external measurements. (a) distance between medial canthi of the eyes (intercanthal distance); (b) distance between the center of both eyes (interpupillary distance); (c) distance from the stop to the external occipital protuberance following the shape of the head (cranial length) (1) lateral view (2) dorsal view; (d) circumference (transverse plane) of the head over the zygomatic arch; (e) length of the mandible (rostral tip of the mandible to the angular process); (f) maximum width of the head at the level of the zygomatic arch (1) rostral view, (2) dorsal view; (g) length of the dorsal aspect of the nose from the planum to the stop (muzzle length).

External measurements were taken from the head of dogs when under general anesthesia by one of the authors (PM) using a tape measure and a caliper. Measurements included:

- Distance between medial canthi of the eyes (intercanthal distance) (Fig 2A);
- Distance between the center of both eyes (interpupillary distance) (Fig 2B);
- Distance from the stop to the external occipital protuberance following the shape of the head (cranial length) (Fig 2C);
- Circumference (transverse plane) of the head over the zygomatic arch (Fig 2D);
- Length of the mandible (rostral tip of the mandible to the angular process) (Fig 2E);
- Maximum width of the head at the level of the zygomatic arch (Fig 2F), and;
- Length of the dorsal aspect of the nose from the planum to the stop (muzzle length) (Fig 2G).

Ratios were calculated for the following measurements:

- Interpupillary distance: intercanthal distance (Ratio 1)
- Circumference of the head over the zygomatic arch: Distance from the stop to the external occipital protuberance following the shape of the head (modified cephalic index 1) (Ratio 2)
- Maximum width of the head at the level of the zygomatic arch: Distance from the stop to the external occipital protuberance following the shape of the head (modified cephalic index 2) (Ratio 3)
- Length of the dorsal aspect of the nose from the planum to the stop (muzzle length): Distance from the stop to the external occipital protuberance following the shape of the head (craniofacial ratio, Ratio 4)
- Length of the dorsal aspect of the nose from the planum to the stop (muzzle length): Length of the mandible (Ratio 5)

### Statistical analysis

Statistical analyses were performed using Microsoft Excel® and R v4.3.1. P-values < 0.050 were regarded as significant. Descriptive statistics are reported. Data were tested for normality using a Kolmogorov-Smirnov test. Consistency between CT-based measurements and manual external measurements of continuous variables of the distance between medial canthi of the eyes (intercanthal distance), the distance between the center of both eyes (interpupillary distance), and the length of the dorsal aspect of the nose from the planum to the stop (muzzle length) was assessed with the ICC (model = two-way random effects, type = consistency, unit = single rating) as these measurements are based on the same anatomical constituents (i.e. the dog’s head for manual external measurements and 3D CT reconstruction of the dog’s head). Correlation between other continuous variables was assessed by determination of the Pearson correlation coefficient. ICC values were interpreted as: < 0.5 = poor; 0.5–0.75 = moderate; 0.75–0.90 = good; > 0.90 = excellent. Pearson correlation coefficients were interpreted as: 0.00–0.30 = no to very weak; 0.30–0.50 = weak; 0.50–0.70 = moderate; 0.70–0.90 = strong; 0.90–1.00 = very strong to perfect.

Two-sample t-tests assuming unequal variances were performed to analyze for differences between CM and SM normal versus abnormal group means of CT measurements, age, and weight. Simple logistic regression analysis was performed on the datasets with manual external measurements and ratios thereof as independent (predictor) variables and CM/SM status (normal versus abnormal) as dependent variables. Variables with a (Wald) p-value of < 0.050 were regarded as significant, compared to a null model. Variables with (Wald) p-values of < 0.2 on univariable modelling were planned to be carried forward to a multiple logistic regression model. The logistic regression model’s goodness-of-fit was assessed using a likelihood ratio test, with a p-value of, <0.050 interpreted as a better fit compared to a null model (i.e. a useful model). McFadden’s R^2^ was calculated to assess the superiority of the model over the null model. For the final model, the area under the receiver operator characteristic (ROC) curve (AUC) was calculated. The optimal cutoff point (according to Youden [25] and cutoff points with optimized sensitivity and specificity were determined.

## Results

### Study population

Ninety-two (92) dogs were included in the study. S1 Table in S1 File includes characteristics of the study population.

### CM and SM prevalence

The prevalence of CM and SM and classification as CM or SM normal versus abnormal are included in contingency tables (S2 Table in S1 File). Of the 92 dogs, 31 (34%) were classified as CM normal (CM0) and 61 (66%) were classified as CM abnormal (CM1 and CM2). Of the 92 dogs, 45 (49%) were classified as SM normal (SM0) and 47 (51%) were classified as SM abnormal (SM1 and SM2).

Age and weight were not significantly different between CM normal (age: 3.1 years (SD 1.6), weight: 3.5 kg (SD 1.1)) versus CM abnormal dogs (age: 3.1 (SD 1.4) years, weight: 3.3 (SD 0.9) kg) or SM normal (age: 2.9 (SD 1.5) years, weight: 3.5 (SD 1.0)) versus SM abnormal dogs (age: 3.2 (SD 1.3) years, weight: 3.2 (SD 1.0) kg) (T-test, p = 0.170–0.909).

### Intra- and interobserver reliability of CT-based measurements

The intra-observer reliability based on ICC was excellent for all CT-based measurements included in the study (ICC 0.91–0.99). The interobserver reliability was good to excellent for all CT-based measurements included in the study (ICC 0.80–0.95).

### Consistency and correlation between CT-based measurements and manual external measurements

Field-of-view did not cover the most rostral extent of the mandible or nose in 20 and 39 dogs, respectively. Therefore, no results were recorded for CT-based measurements of the length of the mandible and the length of the dorsal aspect of the nose from the planum to the stop (muzzle length) for those dogs. This left data for 72 and 53 dogs, respectively, to be included for these measurements.

For manual external measurements of the distance between medial canthi of the eyes (intercanthal distance) and the distance between the center of both eyes (interpupillary distance), results of 22 dogs were not documented. Therefore, measurements of 70 dogs were included for analysis.

Moderate intermodality consistency was identified for the measurements of the length of the dorsal aspect of the nose from the planum to the stop (muzzle length; ICC = 0.647 (95% CI 0.458–0.780, p < 0.001). For the measurements of both the distance between medial canthi of the eyes (intercanthal distance) and the distance between the center of both eyes (interpupillary distance), poor intermodality consistency was found (ICC = 0.314 (95% CI 0.087–0.510, p = 0.004) and ICC = 0.474 (95% CI 0.271–0.637 (p < 0.001)).

A moderate positive correlation was found for the measurement of the length of the mandible (Pearson correlation coefficient = 0.65, p < 0.001). Weak positive correlations were identified for the measurements of the distance from the stop to the external occipital protuberance following the skull borders (CT-based cranial length)/to the external occipital protuberance following the shape of the head (cranial length) (Pearson correlation coefficient = 0.38, p < 0.001) and circumference of the skull/head at the level of the orbital fissure/over the zygomatic arch (Pearson correlation coefficient = 0.38, p < 0.001). A very weak positive correlation was found for the measurement of the maximum width of the skull/head at the level of the orbital fissure/zygomatic arch (Pearson correlation coefficient = 0.26, p = 0.012).

### Differences in CT-based measurements and ratios between CM/SM normal versus abnormal dogs

The mean values for CT-based measurements in CM normal and CM abnormal dogs are included in S3 Table in S1 File. No statistically significant differences were identified for any of the measurements between CM normal versus CM abnormal dogs (p > 0.050). The test result for a difference in length of the mandible between CM abnormal dogs (6.3 cm (SD 0.6 cm)) versus CM normal dogs (6.5 cm (SD 0.5 cm)) neared significance (p = 0.051). None of the ratios were statistically significantly different between the groups (p > 0.050).

The mean values for CT-based measurements in SM normal and SM abnormal dogs are included in S4 Table in S1 File. The length of the mandible was significantly shorter for SM abnormal dogs (6.2 cm (SD 0.5 cm)) compared to SM normal dogs (6.5 cm (SD 0.5 cm))) (p = 0.013). No statistically significant differences were identified for any of the other measurements or ratios between SM normal versus SM abnormal dogs.

### Logistic regression modelling for CM/SM status based on manual external measurements

Simple logistic regression analysis with CM status (abnormal versus normal) as dependent variable yielded no associations to external manual measurements or ratios (Wald p-values of >0.2).

Simple logistic regression analysis with SM status (abnormal versus normal) as dependent variable yielded length of the mandible as the only significant predictive manual external measurement. None of the other external manual measurements or ratios were associated with SM status. Mandible length was moderately correlated with weight (Pearson correlation coefficient = 0.585, p < 0.001).

The logistic regression model with SM status (abnormal versus normal) as dependent variable and length of the mandible as explanatory variable is (eq 1):

ln(oddsSMabnormalversusnormal)=4.1344+−0.05856(lengthofthemandibleinmm)

Where 4.1344 = β0 = intercept (p = 0.041) and -0.05856 = β1 = coefficient = log odds ratio (p = 0.043). Negativity of the coefficient means that a longer mandible is associated with a lower odds of having SM.

The likelihood ratio test indicated that the model was a good fit *(χ2(1) = 4*.*63*, *p = 0*.*031)*. McFadden’s pseudo R^2^ for this model = 0.036, indicating a small overall effect size.

The probability of being classified as SM abnormal (i.e. having SM) based on the length of the mandible, can be predicted as follows (eq 2):

$$Predicted\;probability\;of\;having\;SM=\frac{1}{1+e^{-(4.1344+\;-0.05856(length\;of\;the\;mandible\;in\;mm))}}$$

**Fig 3:**
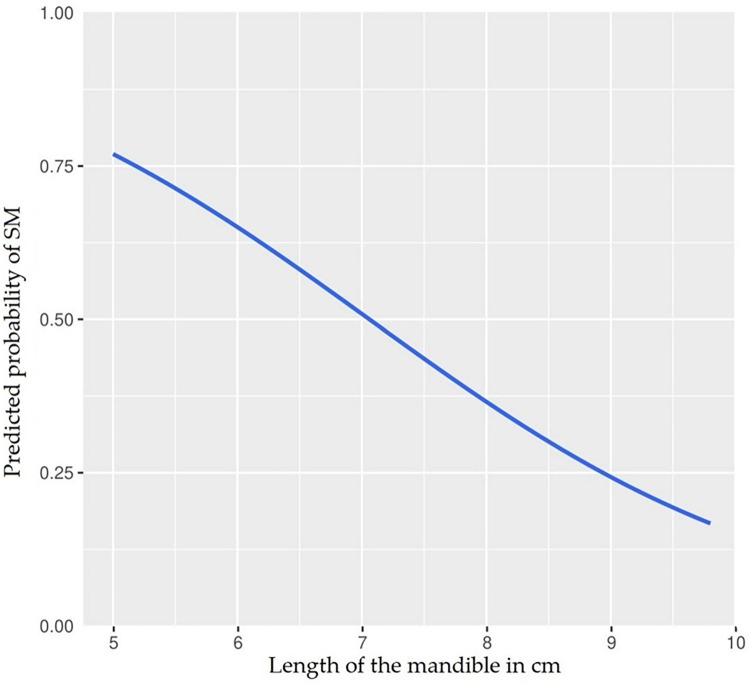
Mandible length to predict the probability of syringomyelia. Plot of the predicted probability function (eq 2) where the length of the mandible in cm is on the x-axis and the probability of having SM is on the y-axis.

Fig 3 shows the plotted graph where the x-axis is the length of the mandible (in cm) and the y-axis is the probability of having SM.

The AUC for the ROC was 0.6326 (CI 95% 0.5247–0.7406). Table 1 lists cutoff values (Youden, and two selections for high sensitivity and high specificity) for mandible length and corresponding sensitivity and specificity with 95% confidence intervals.

**Table 1:** Cutoff values (Youden, high sensitivity and high specificity) for mandible length with sensitivity and specificity.

Cutoff value	Type	Sensitivity % (95% CI^1^)	Specificity % (95% CI^1^)
73 mm	Youden	85 (74–94)	33 (20–47)
58 mm	High sensitivity	96 (89–100)	7 (0–16)
82 mm	High specificity	6 (0–15)	98 (93–100)

^1^Confidence interval

## Discussion

In this study, we compared CT-based measurements and manual external measurements of the skull and head between Pomeranians with and without CM/SM. The results of our study provide important insights into morphometric evaluation of Pomeranians with CM/SM as well as a clinically useful parameter (manual measurement of mandible length) to assess the probability of a dog having CM/SM. Each of our main findings will be discussed in turn in the following paragraphs.

### Consistency and correlation between CT-based measurements and manual external measurements

Consistency and correlation between CT-based measurements and external manual measurements was poor to moderate. These findings could be explained by several reasons.

First, the reliability of manual external measurement was not assessed in this study, nor in a previous study in the CKCS [8]. Manual external measurements are most likely less reliable than the CT-based measurements. Second, several of the manual external measurements were not exactly comparable to the CT-based measurements. This was taken into account in the analysis by using ICC analysis for those measurements (e.g. CT-based cranial length versus the manual external measurement cranial length) and using Pearson correlation coefficient for the others (e.g. intercanthal distance). Still, we cannot exclude imprecision of the manual external measurements influencing the analysis. Third, CT-based measurements were done by using imaging software (i.e. digital) while manual external measurements where done by using a tape measure and calipers (i.e. physical). Fourth, the Pomeranian is a dog with abundant fur and this may impact manual external measurements.

In any case, it is important to realize that manual external measurements do not correlate well with CT-based measurements. This means that the external measurements should not be used as substitutes of CT-based measurements. Still, CT-based and manual external measurement of mandible length showed the highest intermodality correlation (Pearson correlation coefficient = 0.65). As both the CT-based and manual external measurement of mandible length was associated with the presence of SM, this appears to be a robust finding.

### Differences in CT-based measurements and ratios between CM/SM normal versus abnormal dogs

In this study, we did not identify any significant differences between CM normal versus CM abnormal dogs for any of the measurements or ratios included. However, the test result for a difference in length of the mandible between dogs with or without CM approached significance (p = 0.051). Since mandible length was significantly shorter for dogs with SM compared to dogs without SM, and the accepted association between CM and SM, we recommend to consider to include assessment of mandible length in relation to the presence or absence of CM in future studies.

The significantly shorter mandible length as measured on CT images in SM abnormal dogs compared to SM normal dogs was a main finding of this study. This aligned with the significant association of manual external measurement of mandible length.

None of the ratios were statistically significantly different between the groups (p > 0.050). We included 5 different ratios for both the CT-based measurements and manual external measurements. We will discuss each in turn and how they relate to previous studies below.

### Ratio 1

Ratio 1 = Interpupillary distance: intercanthal distance

In our study, we measured interpupillary and medial intercanthal distances because these parameters are often examined in research on human syndromic disorders, which can involve either CM or SM [26–31]. While CM is ubiquitous in CKCS dogs [17, 32–35], its prevalence in Pomeranians was 66% in our study population. We aimed to identify whether these eye-related measurements were associated with CM, rather than SM alone. A prior study on SM in CKCS dogs [8] found no association between similar measurements and SM. Although we did not include all the same measurements, our findings do not indicate a link between interpupillary or medial intercanthal distances and CM/SM in Pomeranians.

### Ratio 2 & 3

Ratio 2 = Circumference of the head over the zygomatic arch: Distance from the stop to the external occipital protuberance following the shape of the head (modified cephalic index 1)

Ratio 3 = Maximum width of the head at the level of the zygomatic arch: Distance from the stop to the external occipital protuberance following the shape of the head (modified cephalic index 2)

These ratios were designed to assess for associations between the modified cephalic index and CM/SM. Previous studies evaluated the reliability of the external measurements and ratios including cephalic index and rostrocaudal doming as indicators of brachycephaly and SM in CKCS [8, 36]. Initially, a positive correlation between these two parameters and SM risk was found, suggesting they could be risk factors. However, it was concluded that these measurements alone are unreliable [36]. The authors argued that visual assessments and ‘other features’ (including those on judges’ checklists and the size of the dog) should be considered for a more accurate evaluation [36]. Our analysis with a modified cephalic index confirmed no significant association with CM or SM, supporting the conclusion that these indices cannot, in isolation, be confidently relied upon to identify at-risk dogs.

We calculated Ratio 2 and Ratio 3 as modified cephalic indices, taking into account doming of the skull by measuring the distance over the top of the skull [8]. We did not find associations with CM or SM for both the CT-based measurements as well as the manual external measurements. These findings are in line with the conclusions of Knowler et al. [36], who stated that these indices cannot be confidently relied upon to identify dogs at risk for CM/SM.

### Ratio 4 & 5

Ratio 4 = Length of the dorsal aspect of the nose from the planum to the stop (muzzle length): Distance from the stop to the external occipital protuberance following the shape of the head (craniofacial ratio)

Ratio 5 = Length of the dorsal aspect of the nose from the planum to the stop (muzzle length): Length of the mandible

Both these ratios are discussed alongside each other, as both assess relative muzzle length in relation to different parts of the skull anatomy.

The craniofacial ratio has been described by Packer et al. as a measure of relative muzzle length [35, 37, 38]. Lower ratios (relatively shorter muzzles) characterize brachycephalic dogs compared to non-brachycephalic dogs. A lower craniofacial ratio has been associated with brachycephalic obstructive airway syndrome (BOAS) as well as ocular disorders [9, 37]. In our study, we found no association with CM/SM for both the absolute measurements as well as the craniofacial ratio.

Muzzle length was not associated with risk of SM in a previous study [6]. Indeed, muzzle length may be misleading when assessing head conformation of dogs in relation to CM/SM [36], i.e. dogs with long muzzles may still have CM/SM. However, as mandible length was not accounted for in those studies, we were interested to see if the muzzle length to mandible length ratio would yield any significant results. While we did identify a difference in mandible length between SM normal and SM abnormal dogs, Ratio 4 was not significantly different between SM normal or abnormal dogs.

In conjunction with the results of the previous CKCS studies [8, 36], our results indicate that muzzle length measurements for the assessments of the risk of SM in dogs is not particularly relevant.

### Logistic regression modelling for CM/SM status based on manual external measurements

As mentioned above, SM abnormal dogs had a significantly shorter mandible length based on CT measurements compared to SM normal dogs. We also identified a significant association between the external manual measurement of mandible length and SM status. SM abnormal dogs had significantly shorter mandibles than SM normal dogs. None of the other manual external measurements was associated with CM or SM status.

Measurement of mandible length was not included in a previous study in the CKCS that studied manual external measurements in relationship to SM [8]. We included measurements of mandible length for two main reasons. First, compared to the muzzle or maxilla, the mandible is a less complex piece of the skull. The bone itself articulates with but does not directly attach to other bones. Using externally palpable anatomical features (i.e. rostral tip and the angular process), it lends itself excellently to manual external measurement of its length. Second, a short mandible is one of the features of brachycephaly and brachycephaly is a characteristic of most dog breeds reported to be affected by CM/SM [39, 40].

Changes to the position and length of the mandible in brachycephalic dogs have been studied and extensively described and reviewed in literature [40–43]. Importantly, it has been shown that cranial and mandible morphological characteristics are associated [43]. Pomeranians are described as a breed with “allometric brachycephaly” [40]. Allometry refers to ‘*changes of biological variables*, *e*.*g*., *shape of an organ/structure such as the skull*, *correlated with changes of the size of the same organ/structure or overall body size*’ [40]. This group of brachycephalic dogs includes most small or toy breed varieties, such as the Chihuahua as well. Compared to other types of brachycephaly (“bulldog” and “katantognathic” types), these breeds show no consistent angular change facial bones relative to bones of the cranium compared with larger varieties such as the CKCS [3]. “Airorhynchy” is defined as ‘*dorsal rotation/upward tilting of the palate relative to the cranial base*’ or in simpler, less precise terms: ‘*upward tilting of the snout*’ [39]. Sometimes, this is regarded as synonymous to brachycephaly. This particular feature is recognized in the poster breed for CM/SM in dogs, the CKCS [3, 7, 8, 44]. However, Pomeranians and the other breeds referred to as allometric brachycephalic dogs do not show this feature. Differences in cranial suture or synchondroses closure have been identified in other brachycephalic dog breeds, including the CKCS [45, 46]. Such studies have not been performed specifically in the Pomeranian dog breed. It is likely that the pathophysiology of CM/SM in Pomeranians differs from those breeds in particular, while it may be more comparable with other allometric brachycephalic dogs, like the Chihuahua. Future studies that assess the relationship between mandible length and risk of SM may provide more information of the validity of the manual external measurement to predict the probability of SM in dogs of those breeds.

A moderate correlation between mandible length and weight in our study does give rise to the consideration that the association between mandible length and SM is partly explained by size of the dog. Larger dogs (higher body weight) have longer mandibles. Indeed, this comes a no surprise, as many scientific publications (recently reviewed) have already provided solid evidence that this allometry exists in most vertebrates including dogs [41]. Similarly, it has been documented extensively that the brain of smaller animals is proportionally larger compared to larger animals, a biological rule referred to as ‘Haller’s rule’ [40, 47]. Mismatches or compensatory changes with dog breeds or family of dogs of a particular breed may play a role in the pathophysiology of CM/SM, particularly allometric brachycephalic dogs, like the Pomeranian.

As body condition score is not taken into account in absolute weights and size is determined by other factors than weight, it is not surprising that there was only a moderate correlation. Correlations of manual measurements with size or body weight were not reported in a CKCS study [8]. In our study, there were also small dogs with low body weight and relatively long mandibles and vice versa. Nonetheless, taking into account size of the dog in the clinical evaluation of the risk of SM would be prudent [36].

### Application of the model and cutoff values

We must thoroughly evaluate the results of our logistic regression model before applying it in clinical practice or breeding programs. For the single statistically significant variable of mandibular length measured manually (externally), the model’s McFadden’s pseudo R^2^ is low. This can be interpreted as an overall small effect size, i.e. the model’s ability to predict the SM status based on the length of the mandible is weak. This is not surprising, as it is unlikely that any of the studied variables would account for all the variance in the presence or absence of SM. In other words, the model cannot be expected to explain or correctly predict the presence or absence of SM in all cases. This suggests the logical notion that our model does not include all variables that explain the occurrence of SM in Pomeranians. Future studies including other variables of different natures or other types of measurements may yield more variables of interest.

The value of this particular model reported here should be verified by future studies. Practically, having an easy way to determine the probability of SM in a Pomeranian (i.e. manually measuring the length of the mandible) is of great value. When MRI examinations are not possible, assessing owner-reported clinical signs [21] and measuring the length of the mandible can help to determine the likelihood of a dog having SM. However, MRI is required for a formal diagnosis of SM. Using clinical signs or mandible length measurements alone is not recommended for f.i. selection of dogs for breeding programs.

Application of the cutoff values mentioned in Table 1 should be considered in light of the above considerations. Having a mandible length shorter than 58 mm means the model judges the probability of that Pomeranian being SM abnormal is high. Conversely, a mandible length of 82 mm or longer means the model assesses the dog’s probability of being SM normal is high. However, the number of dogs included in the construction of this model that have such short or long mandible was low. In clinical practice, dogs with mandible lengths in between these extreme cutoff values would likely account for the vast majority. The Youden cutoff value has a lower sensitivity and very low specificity and its clinical applicability is questionable. Therefore, these cutoff values should be taken as illustration that a shorter mandible increases the probability of SM compared to a longer mandible. Strict application of or relying solely on the cutoff values is inadvisable, as, again, diagnosis of SM is based on MRI.

### Limitations

The reliability of manual external measurements has not been assessed in this or previous studies [8, 36]. Future studies assessing the reliability of manual external measurements, especially the measurement of mandible length, may provide important information on the clinical applicability and value of these measurements. Of consideration for such studies would be to include photograph-based [8, 36], computerized measurements from digital images (i.e. photographs or 3D videographic scans), or radiograph-based measurements, and compare these to manual measurements. However, the benefits of manual external measurements include that it is simple, cost-effective, and requires no other materials than calipers and tape measures.

While this study includes more dogs than many other studies on CM/SM in dogs, the number of included dogs is still limited and some statistically non-significant findings might be due to lack of power. Indeed, future studies, with more dogs, or even the same number of dogs, will undoubtedly yield different absolute results, surely with respect to model building. This emphasizes our point that the model based on mandible length measurement should not be used in isolation to guide clinical practice or breeding program selections.

## Conclusions

Manual external measurement of mandible length can be easily performed without sedation or anesthesia in most dogs. The presence or absence of owner reported clinicals signs in addition to the measurement of the length of the mandible can help to determine the probability of a Pomeranian having SM. MRI is still required to formally diagnose SM.

## Supporting information

S1 File **Table S1. Characteristics of the study population**.^1^ Median and interquartile range.**Table S2. Contingency table including numbers and percentages (of total) of included dogs’ CM and SM classifications (grey = abnormal, white = normal).**.CM, Chiari-like malformation; SM, syringomyelia.**Table S3. Group means for CT-based measurements for CM normal versus abnormal.**.^1^Standard deviation, * based on 72 pairs of measurements, ** based on 53 pairs of measurementsAll measurements are in centimeters. Ratio 1: Interpupillary distance: intercanthal distance; Ratio 2: Circumference of the skull at the level of the orbital fissure: Distance from the stop to the external occipital protuberance following the skull borders (modified cephalic index 1); Ratio 3: Maximum width of the skull at the level of the orbital fissure: Distance from the stop to the external occipital protuberance following the skull borders (modified cephalic index 2); Ratio 4: Length of the dorsal aspect of the nose from the planum to the stop (muzzle length): Distance from the stop to the external occipital protuberance following the skull borders (CT-based craniofacial ratio); Ratio 5: Length of the dorsal aspect of the nose from the planum to the stop: Length of the mandible.**Table S4. Group means for CT-based measurements for SM normal versus abnormal.**.^1^Standard deviation, * based on 72 pairs of measurements, ** based on 53 pairs of measurementsAll measurements are in centimeters. Ratio 1: Interpupillary distance: intercanthal distance; Ratio 2: Circumference of the skull at the level of the orbital fissure: Distance from the stop to the external occipital protuberance following the skull borders (modified cephalic index 1); Ratio 3: Maximum width of the skull at the level of the orbital fissure: Distance from the stop to the external occipital protuberance following the skull borders (modified cephalic index 2); Ratio 4: Length of the dorsal aspect of the nose from the planum to the stop (muzzle length): Distance from the stop to the external occipital protuberance following the skull borders (CT-based craniofacial ratio); Ratio 5: Length of the dorsal aspect of the nose from the planum to the stop: Length of the mandible.
(PDF)

S2 File Table S5. Data file.
(PDF)
