# Supplementary material for: CT-based and manual external skull measurements for Chiari-like malformation and syringomyelia in Pomeranians
Source: PLoS One. 2024 Nov 25;19(11):e0313203. doi: 10.1371/journal.pone.0313203 (PMC11588269; doi:10.1371/journal.pone.0313203)
Supplement: S1 File — Table S1. Characteristics of the study population.1 Median and interquartile range.Table S2. Contingency table including numbers and percentages (of total) of included dogs’ CM and SM classifications (grey = abnormal, white = normal)..CM, Chiari-like malformation; SM, syringomyelia.Table S3. Group means for CT-based measurements for CM normal versus abnormal..1Standard deviation, * based on 72 pairs of measurements, ** based on 53 pairs of measurementsAll measurements are in centimeters. Ratio 1: Interpupillary distance: intercanthal distance; Ratio 2: Circumference of the skull at the level of the orbital fissure: Distance from the stop to the external occipital protuberance following the skull borders (modified cephalic index 1); Ratio 3: Maximum width of the skull at the level of the orbital fissure: Distance from the stop to the external occipital protuberance following the skull borders (modified cephalic index 2); Ratio 4: Length of the dorsal aspect of the nose from the planum to the stop (muzzle length): Distance from the stop to the external occipital protuberance following the skull borders (CT-based craniofacial ratio); Ratio 5: Length of the dorsal aspect of the nose from the planum to the stop: Length of the mandible.Table S4. Group means for CT-based measurements for SM normal versus abnormal..1Standard deviation, * based on 72 pairs of measurements, ** based on 53 pairs of measurementsAll measurements are in centimeters. Ratio 1: Interpupillary distance: intercanthal distance; Ratio 2: Circumference of the skull at the level of the orbital fissure: Distance from the stop to the external occipital protuberance following the skull borders (modified cephalic index 1); Ratio 3: Maximum width of the skull at the level of the orbital fissure: Distance from the stop to the external occipital protuberance following the skull borders (modified cephalic index 2); Ratio 4: Length of the dorsal aspect of the nose from the planum to the stop (muzzle length): Dista [file pone.0313203.s001.pdf]

Supporting Information

CT-based and Manual External Skull Measurements for Chiari-like Malformation and Syringomyelia in Pomeranians

Koen Santifort [1,2,3*], Sophie Bellekom [2], Ines Carrera [4] and Paul Mandigers [1,3*]

[1] IVC Evidensia Referral Hospital Arnhem, Arnhem, Netherlands; koen.santifort@evidensia.nl

[2] IVC Evidensia Referral Hospital Hart van Brabant, Waalwijk, Netherlands

[3] Expertise Centre of Genetics, Department of Clinical Sciences, Faculty of Veterinary Medicine, Utrecht University, Utrecht, Netherlands

[4] Vet Oracle Teleradiology, Norfolk, United Kingdom

* Corresponding author:

koen.santifort@evidensia.nl (KS), p.j.j.mandigers@uu.nl (PM)

Table S1. Characteristics of the study population.

**Total study population**	**99 (100%)**
**Sex**	
Female	41 (45%)<br>40 intact, 1 neutered
Male	51 (55%)<br>45 intact, 6 neutered
**Age[1]**	2.9 years (2.0 – 3.8)
**Weight[1]**	3.3 kg (2.6 – 4.0)

[1] Median and interquartile range

Table S2. Contingency table including numbers and percentages (of total) of included dogs' CM and SM classifications (grey = abnormal, white =normal).

Classification	SM0	SM1	SM2	Total
**CM0**	17 (18%)	11 (12%)	3 (3%)	31 (34%)
**CM1**	26 (28%)	18 (20%)	12 (13%)	56 (61%)
**CM2**	2 (2%)	3 (3%)	0 (0%)	5 (5%)
**Total**	45 (49%)	32 (35%)	15 (16%)	92 (100%)

CM, Chiari-like malformation; SM, syringomyelia.

Table S3. Group means for CT-based measurements for CM normal versus abnormal.

Item	CM normal	CM abnormal	
	**Mean (SD[1])**	**Mean (SD)**	**P-value**
Distance between medial canthi of the eyes	3.2 (0.3)	3.3 (0.3)	0.244
Distance between the center of both eyes	4.5 (0.3)	4.6 (0.3)	0.362
Distance from the stop to the external occipital protuberance following the skull borders (CT-based cranial length)	8.5 (0.4)	8.4 (0.4)	0.206
Circumference of the skull at the level of the orbital fissure	13.7 (0.4)	13.7 (0.6)	0.856
Length of the mandible (rostral tip of the mandible to the angular process)*	6.5 (0.5)	6.3 (0.6)	0.051
Maximum width of the skull at the level of the orbital fissure	4.4 (0.2)	4.4 (0.2)	0.448
Length of the dorsal aspect of the nose from the planum to the stop**	3.0 (0.5)	3.1 (0.6)	0.644
Ratio 1	1.40 (0.10)	1.39 (0.08)	0.455

Ratio 2	1.60 (0.06)	1.63 (0.07)	0.082
Ratio 3	0.52 (0.02)	0.53 (0.03)	0.101
Ratio 4	0.36 (0.05)	0.37 (0.07)	0.493
Ratio 5	0.47 (0.07)	0.49 (0.08)	0.2925

[1]Standard deviation, * based on 72 pairs of measurements, ** based on 53 pairs of measurements

All measurements are in centimeters. Ratio 1: Interpupillary distance : intercanthal distance; Ratio 2: Circumference of the skull at the level of the orbital fissure : Distance from the stop to the external occipital protuberance following the skull borders (modified cephalic index 1); Ratio 3: Maximum width of the skull at the level of the orbital fissure : Distance from the stop to the external occipital protuberance following the skull borders (modified cephalic index 2); Ratio 4: Length of the dorsal aspect of the nose from the planum to the stop (muzzle length) : Distance from the stop to the external occipital protuberance following the skull borders (CT-based craniofacial ratio); Ratio 5: Length of the dorsal aspect of the nose from the planum to the stop : Length of the mandible.

Table S4. Group means for CT-based measurements for SM normal versus abnormal.

**Item**	**SM normal**	**SM abnormal**	
	**Mean (SD[1])**	**Mean (SD)**	**P-value**
Distance between medial canthi of the eyes	3.3 (0.3)	3.2 (0.3)	0.3287
Distance between the center of both eyes	4.6 (0.3)	4.5 (0.3)	0.1660
Distance from the stop to the external occipital protuberance following the skull borders (CT based-cranial length)	8.5 (0.4)	8.5 (0.4)	0.9744
Circumference of the skull at the level of the orbital fissure	13.7 (0.5)	13.7 (0.6)	0.5121
**Length of the mandible (rostral tip of the mandible to the angular process)***	**6.5 (0.5)**	**6.2 (0.5)**	**0.0125**
Maximum width of the skull at the level of the orbital fissure	4.4 (0.2)	4.4 (0.2)	0.5839

Length of the dorsal aspect of the nose from the planum to the stop**	3.1 (0.6)	3.1 (0.5)	0.8238
Ratio 1	1.40 (0.08)	1.39 (0.08)	0.6940
Ratio 2	1.62 (0.07)	1.62 (0.07)	0.5881
Ratio 3	0.52 (0.03)	0.53 (0.03)	0.5238
Ratio 4	0.36 (0.07)	0.37 (0.06)	0.758
Ratio 5	0.47 (0.08)	0.51 (0.06)	0.0793

[1]Standard deviation, * based on 72 pairs of measurements, ** based on 53 pairs of measurements

All measurements are in centimeters. Ratio 1: Interpupillary distance : intercanthal distance; Ratio 2: Circumference of the skull at the level of the orbital fissure : Distance from the stop to the external occipital protuberance following the skull borders (modified cephalic index 1); Ratio 3: Maximum width of the skull at the level of the orbital fissure : Distance from the stop to the external occipital protuberance following the skull borders (modified cephalic index 2); Ratio 4: Length of the dorsal aspect of the nose from the planum to the stop (muzzle length) : Distance from the stop to the external occipital protuberance following the skull borders (CT-based craniofacial ratio); Ratio 5: Length of the dorsal aspect of the nose from the planum to the stop : Length of the mandible.
