# Supplementary material for: CT-based and manual external skull measurements for Chiari-like malformation and syringomyelia in Pomeranians
Source: PLoS One. 2024 Nov 25;19(11):e0313203. doi: 10.1371/journal.pone.0313203 (PMC11588269; doi:10.1371/journal.pone.0313203)
Supplement: S2 File — Data file. (PDF) [file pone.0313203.s002.pdf]

INCLUSION NUMBER	Sex	Age	Body weight	CM CLASSIFICATION	CM NORMAL 0 ABNORMAL 1	SM CLASSIFICATION	SM normal 0 or abnormal 1	CT
1	M	2.96	2.4	1	1	1	1	
2	M	2.89	2.2	1	1	2	1	
3	F	2.19	1.6	1	1	1	1	
4	MN	3.79	2.3	2	1	1	1	
5	M	3.66	2.9	1	1	2	1	
6	M	3.52	4.3	2	1	1	1	
7	M	1.84	3.1	1	1	1	1	
8	M	2.38	3.3	0	0	1	1	
9	M	3.08	3.4	1	1	1	1	
10	F	5.23	3.0	1	1	1	1	
11	F	1.13	2.7	0	0	2	1	
12	F	2.71	2.2	1	1	1	1	
13	M	3.93	4.8	0	0	0	0	
14	F	2.84	2.4	1	1	1	1	
15	F	5.11	4.1	2	1	1	1	
16	M	2.36	3.4	0	0	1	1	
17	F	2.90	4.0	1	1	0	0	
18	M	3.60	3.3	1	1	1	1	
19	F	6.80	2.8	1	1	0	0	
20	M	7.46	6.8	0	0	1	1	
21	M	4.33	3.8	1	1	2	1	
22	MN	2.46	4.3	1	1	0	0	
23	F	2.30	2.8	1	1	2	1	
24	M	6.64	4.1	1	1	0	0	
25	M	3.02	4.3	0	0	1	1	
26	F	4.05	2.4	0	0	0	0	
27	M	1.79	3.4	1	1	2	1	
28	F	3.07	3.8	1	1	0	0	
29	M	1.88	1.6	1	1	1	1	
30	F	1.96	2.2	0	0	0	0	
31	M	3.11	3.4	0	0	1	1	
32	F	1.91	2.5	1	1	0	0	
33	M	1.11	2.4	0	0	2	1	
34	M	3.37	2.9	1	1	0	0	
35	M	3.99	3.6	0	0	0	0	
36	MN	5.36	5.7	1	1	0	0	
37	F	2.11	2.9	0	0	0	0	
38	F	2.29	2.5	1	1	0	0	
39	M	3.76	3.9	0	0	1	1	
40	M	1.88	3.5	1	1	1	1	
41	F	3.48	3.5	1	1	0	0	
42	M	2.96	3.5	1	1	1	1	
43	M	3.22	2.4	1	1	2	1	
44	M	3.34	4.4	0	0	0	0	
45	M	1.05	2.4	0	0	1	1	
46	F	1.15	2.7	2	1	0	0	
47	F	4.51	2.6	0	0	2	1	
48	F	1.29	2.3	1	1	0	0	
49	F	5.09	3.2	1	1	0	0	
50	M	5.65	3.8	1	1	2	1	
51	M	2.31	4.7	0	0	0	0	
52	M	2.18	5.0	1	1	1	1	
53	M	4.91	5.3	1	1	1	1	
54	M	1.71	3.3	0	0	0	0	
55	M	2.93	4.2	0	0	0	0	
56	FN	4.19	3.0	1	1	1	1	
57	F	3.76	2.8	1	1	1	1	
58	F	2.87	2.0	0	0	0	0	
59	F	2.06	3.3	1	1	0	0	
60	M	1.24	3.5	1	1	0	0	
61	F	2.90	2.7	1	1	1	1	
62	M	2.94	3.1	0	0	1	1	
63	F	1.16	2.6	1	1	0	0	
64	M	5.06	3.9	1	1	2	1	
65	M	3.21	2.3	0	0	0	0	
66	M	3.21	1.9	1	1	1	1	
67	F	3.49	3.6	1	1	1	1	
68	M	3.25	4.0	1	1	0	0	
69	F	1.97	3.9	1	1	0	0	
70	M	2.93	3.7	1	1	2	1	
71	M	5.83	3.3	0	0	0	0	
72	F	2.00	2.6	1	1	0	0	
73	F	2.08	2.6	1	1	2	1	
74	M	2.56	5.9	1	1	0	0	
75	M	3.29	3.2	1	1	0	0	
76	F	2.19	3.2	1	1	2	1	
77	M	1.61	5.7	0	0	0	0	
78	M	1.16	3.1	1	1	0	0	
79	F	4.00	4.3	1	1	2	1	
80	F	1.20	2.4	0	0	0	0	
81	F	5.61	4.0	0	0	1	1	
82	F	6.28	4.0	0	0	0	0	
83	F	2.26	3.1	1	1	0	0	
84	F	2.18	2.2	1	1	0	0	
85	MN	1.63	4.6	2	1	0	0	
86	F	1.60	2.8	0	0	1	1	
87	F	2.33	3.4	0	0	0	0	
88	F	3.22	2.9	0	0	1	1	
89	M	3.43	3.6	0	0	0	0	
90	MN	1.26	4.1	1	1	0	0	
91	F	2.00	4.0	1	1	0	0	
92	MN	4.88	4.5	1	1	0	0	

distance between medial canthi of the eyes (3D reconstruction) (cm 2 decimals)	distance between the center of both eyes (3D reconstruction) (cm 2 decimals)
3.37	4.94
2.93	4.29
2.95	3.86
3.16	4.49
3.22	4.53
2.98	4.24
3.38	4.61
3.54	4.76
3.56	5.23
3.42	4.53
2.90	4.41
3.03	4.35
3.36	5.03
3.54	4.62
2.94	4.15
3.30	4.88
2.94	4.36
3.42	4.75
3.17	4.38
3.96	5.29
3.78	5.00
3.73	5.14
3.01	4.64
3.05	4.33
3.03	4.40
2.72	4.05
3.34	4.74
3.36	4.34
2.80	4.00
3.06	3.86
3.34	4.16
3.31	4.22
3.13	4.23
3.43	4.76
3.07	4.19
3.65	5.19
3.35	4.68
3.23	4.67
3.62	4.44
3.10	4.07
3.41	4.69
2.82	4.28
2.78	4.18
3.39	4.55
3.06	4.30
3.44	4.32
3.69	4.68
3.18	4.79
3.06	4.43
3.40	4.52
3.17	4.52
3.37	4.65
3.44	4.52
3.17	4.64
2.88	4.29
2.90	4.01
2.81	4.00
2.96	3.95
3.20	4.66
3.54	4.51
3.11	4.51
3.89	4.72
2.73	4.24
3.10	4.54
3.24	4.77
3.40	4.79
3.74	4.80
3.68	5.01
3.44	4.91
3.41	4.49
3.04	4.78
3.61	4.36
3.16	4.19
3.98	5.17
3.44	5.04
3.21	4.28
3.10	4.35
3.03	4.17
3.30	4.45
3.27	4.39
3.01	4.59
2.99	4.47
3.67	4.88
3.19	4.60
3.57	5.17
2.77	4.17
3.42	4.58
2.93	4.50
3.13	4.56
3.43	4.83
3.37	4.57
3.73	4.81

distance from the stop to the external occipital protuberance following the skull borders (sagittal reconstruction) (cm 2 decimals)
9.63
8.07
8.14
7.47
8.28
8.06
7.89
8.72
9.21
8.37
8.46
8.57
9.68
8.62
8.93
8.81
8.52
8.53
8.54
8.86
8.12
8.97
8.43
8.42
9.25
8.99
8.12
9.26
7.65
8.39
8.82
8.36
8.84
7.57
8.64
8.50
7.70
8.28
8.07
8.27
8.42
8.37
8.57
8.11
8.43
8.37
8.44
8.31
8.35
9.23
8.46
8.63
8.31
8.53
8.33
8.09
8.33
8.12
8.85
8.53
8.47
8.74
8.24
8.00
8.53
8.35
8.61
8.92
8.78
8.00
8.00
8.13
7.84
8.40
8.49
8.96
8.29
7.82
8.92
8.15
8.42
8.54
8.41
8.01
9.11
7.96
8.99
8.87
8.81
8.56
8.28
8.80

circumference of the skull at the level of the orbital fissure (transverse) (cm 2 decimals)	length of the mandible (rostral tip of the mandible to the condylar process) (3D reconstruction) (cm 2 decimals)
15.30	5.75
13.59	5.89
14.02	XXX
12.93	5.57
13.08	5.63
13.51	6.07
13.97	6.22
14.59	XXX
14.98	6.03
14.09	6.77
13.88	XXX
13.54	5.51
14.61	6.86
13.61	6.16
13.36	6.59
13.94	6.75
13.17	6.54
12.99	6.36
13.48	5.80
13.42	7.27
13.44	5.68
13.53	7.37
14.37	5.66
13.85	6.12
13.98	6.03
13.64	XXX
13.33	XXX
13.66	6.62
13.04	5.20
13.59	6.35
13.95	6.31
13.63	5.98
13.61	6.21
14.24	6.15
13.11	6.66
13.74	XXX
12.73	5.84
14.19	6.35
13.31	6.27
13.36	XXX
13.56	XXX
12.64	5.87
13.78	XXX
13.32	XXX
13.31	XXX
13.67	XXX
13.85	XXX
13.14	XXX
13.51	6.03
14.22	6.16
13.47	6.88
14.27	6.67
13.57	7.73
13.79	7.59
13.79	7.03
13.27	XXX
13.12	5.66
13.14	5.74
14.04	XXX
13.49	6.30
13.49	5.90
13.43	XXX
13.01	6.11
12.73	6.42
13.64	6.44
14.32	6.21
14.25	XXX
14.07	6.20
14.79	7.39
12.64	6.02
13.48	6.81
14.23	6.60
13.20	5.79
14.00	7.60
13.60	6.78
14.37	6.16
14.00	6.55
13.41	6.13
13.57	7.35
13.25	5.52
13.43	6.59
13.99	7.19
13.56	5.55
13.60	6.25
14.39	XXX
13.41	5.67
14.51	6.99
13.49	6.69
14.42	6.60
14.57	7.07
13.57	6.72
14.02	XXX

maximum width of the skull at the level of the orbital fissure (cm 2 decimals)	length of the dorsal aspect of the nose from the planum to the stop (cm 2 decimals)
5.07	3.13
4.49	2.58
4.53	XXX
4.11	XXX
4.04	2.86
4.45	XXX
4.57	2.78
4.70	XXX
4.83	3.09
4.64	3.73
4.39	XXX
4.21	2.98
4.72	XXX
4.44	XXX
4.32	XXX
4.58	3.27
4.32	3.38
4.33	XXX
4.46	3.24
4.08	XXX
4.44	3.31
4.37	4.02
4.97	2.61
4.56	2.87
4.66	XXX
4.37	XXX
4.33	XXX
4.33	2.77
4.19	2.32
4.42	XXX
4.61	3.41
4.41	3.71
4.38	3.07
4.63	1.93
4.14	XXX
4.41	XXX
4.16	XXX
4.62	2.76
4.37	3.56
4.30	XXX
4.33	XXX
4.07	2.69
4.40	XXX
4.16	XXX
4.33	XXX
4.46	XXX
4.58	XXX
4.23	XXX
4.49	3.28
4.83	3.13
4.32	XXX
4.62	3.85
4.40	4.32
4.41	XXX
4.44	3.50
4.33	XXX
4.24	2.41
4.14	2.77
4.52	XXX
4.42	XXX
4.47	4.03
4.38	XXX
4.14	3.00
4.30	3.25
4.39	XXX
4.54	2.93
4.55	XXX
4.34	XXX
4.91	3.10
4.14	3.25
4.11	XXX
4.70	3.14
4.33	2.70
4.54	4.88
4.18	2.71
4.75	2.82
4.50	3.02
4.38	2.44
4.14	XXX
4.16	2.74
4.29	3.35
4.43	2.48
4.21	1.70
4.32	XXX
4.56	XXX
4.47	1.98
4.77	2.74
4.39	3.54
4.66	2.93
4.73	3.69
4.30	3.49
4.45	XXX

Manual	Distance between medial canthi of the eyes (cm one decimal)
	XXX
	XXX
	XXX
	2.8
	2.8
	XXX
	2.4
	3.0
	3.5
	2.5
	XXX
	XXX
	3.1
	2.4
	2.4
	3.6
	3.0
	3.1
	2.6
	3.2
	2.8
	3.2
	2.9
	2.8
	2.8
	2.5
	3.0
	3.0
	2.7
	2.6
	3.2
	3.1
	2.7
	3.1
	2.8
	3.2
	XXX
	XXX
	2.5
	XXX
	2.9
	3.0
	2.9
	2.3
	XXX
	2.6
	3.0
	2.3
	2.7
	XXX
	XXX
	3.2
	2.5
	3.3
	2.7
	XXX
	XXX
	2.1
	XXX
	XXX
	2.8
	XXX
	2.5
	3.0
	2.6
	2.1
	2.9
	3.6
	3.0
	2.8
	XXX
	XXX
	2.4
	XXX
	2.8
	2.8
	2.5
	2.7
	2.7
	2.7
	XXX
	2.8
	3.2
	XXX
	2.3
	2.8
	2.9
	2.6
	2.2
	3.0
	2.8
	2.8

Distance between the center of both eyes (cm one decimal)	Distance from the stop to the dorsal base of the neck following the shape of the head (cm one decimal)
XXX	11.5
XXX	10.0
XXX	10.0
4.3	10.5
4.8	11.0
XXX	10.0
4.8	11.0
4.7	11.0
5.1	12.0
4.2	11.0
XXX	10.0
XXX	11.5
5.2	11.5
4.7	10.3
3.7	9.0
5.0	12.0
4.8	11.0
4.3	10.0
4.3	10.0
5.0	12.5
4.7	10.5
4.5	11.5
4.4	9.5
5.2	10.5
5.0	12.0
4.2	9.5
4.7	10.0
4.9	11.5
4.5	10.0
4.6	11.0
4.5	11.0
4.6	9.5
4.3	10.5
5.1	10.0
4.3	10.5
5.4	13.0
XXX	10.0
XXX	12.0
4.4	9.5
XXX	10.2
4.6	9.5
4.7	10.5
4.7	11.5
5.2	10.5
XXX	10.5
5.1	12.0
4.7	11.0
4.4	9.5
4.5	11.5
XXX	10.5
XXX	11.0
5.2	12.0
4.5	11.0
5.0	11.0
4.6	11.0
XXX	10.5
XXX	12.0
4.2	9.5
XXX	10.0
XXX	11.5
4.3	9.5
XXX	11.0
4.2	10.0
4.6	9.5
4.7	12.0
4.7	10.5
4.9	10.5
5.3	11.0
4.4	11.0
4.7	10.0
XXX	10.5
XXX	11.5
4.1	8.5
XXX	12.5
4.6	12.0
4.7	10.0
4.5	10.0
4.5	10.0
4.6	10.5
4.7	9.0
XXX	9.0
4.7	10.5
4.8	10.0
XXX	10.0
4.9	11.3
4.5	9.5
4.7	10.5
4.8	10.5
4.7	10.0
4.7	10.0
4.4	10.0
4.4	11.5

Circumference of the head over the zygomatic arch at the level of the orbital fissure (cm one decimal)	Length of the mandible (rostral tip of the mandible to the condylar process) (cm one decimal)
26.0	6.0
22.0	7.0
21.0	5.5
21.0	6.0
23.0	7.0
24.0	6.0
22.0	7.0
25.5	7.0
25.5	7.0
23.0	7.5
22.0	7.0
21.0	6.0
26.0	8.0
20.5	5.5
25.0	5.0
25.0	7.0
22.5	7.0
23.0	7.0
22.0	6.5
28.5	9.5
25.5	7.0
27.0	8.0
23.0	6.3
26.0	7.0
24.5	7.0
22.0	6.0
24.5	7.5
24.0	7.0
20.5	6.5
23.0	7.0
24.5	7.5
22.5	5.0
23.0	6.5
24.0	7.0
24.0	7.0
30.0	8.5
23.0	6.5
24.0	7.0
22.0	7.0
24.0	7.0
24.0	7.0
24.0	6.5
23.5	7.0
23.5	7.0
21.5	7.2
24.0	7.0
23.0	6.0
22.7	6.7
21.0	6.5
26.0	7.0
25.0	7.5
28.0	7.0
26.5	8.0
24.0	8.0
25.0	8.0
24.0	6.0
22.0	6.5
21.0	6.0
23.0	7.0
24.0	8.0
23.5	7.0
25.0	9.0
21.5	7.0
24.0	7.0
22.5	7.0
21.5	7.0
25.0	7.0
26.0	7.0
24.5	7.0
25.0	7.0
23.5	7.0
24.0	7.5
21.0	6.0
29.0	9.0
26.0	7.5
25.6	6.8
26.0	7.0
24.0	6.5
24.0	7.5
21.5	6.0
24.0	6.0
24.0	7.0
23.5	6.5
21.5	7.0
27.0	9.8
22.5	6.5
25.0	7.0
22.5	7.0
23.5	7.3
26.5	7.5
24.7	8.0
28.2	8.0

Maximum width of the head at the level of the zygomatic arch (cm one decimal)	Length of the dorsal aspect of the nose from the planum to the stop (cm one decimal)
7.5	2.5
6.6	3.0
6.0	1.5
6.5	2.5
6.7	2.5
6.7	1.7
7.2	2.8
8.2	3.7
7.2	2.9
6.7	3.0
6.7	3.0
6.3	2.0
8.2	3.3
6.3	2.5
7.5	2.8
7.1	3.0
7.0	3.3
7.4	3.0
6.4	3.5
6.9	3.0
7.2	4.5
7.5	3.6
6.4	2.3
7.4	2.9
7.6	2.5
6.0	3.0
7.1	3.8
6.7	2.5
6.0	3.1
6.6	2.9
7.5	3.0
5.9	3.1
6.9	3.2
7.3	2.2
7.3	2.7
8.0	3.5
6.5	2.0
7.5	3.0
7.0	3.2
7.1	3.2
6.9	3.0
6.7	2.5
7.3	2.5
7.1	3.0
7.0	3.2
7.2	3.0
6.7	3.2
5.8	2.9
6.7	3.0
7.2	3.0
7.1	3.5
8.7	3.5
7.5	4.5
7.0	3.5
7.5	3.7
6.8	2.0
6.7	2.6
6.4	3.0
7.1	3.5
7.3	3.5
6.6	3.0
8.2	4.5
6.7	3.0
7.8	3.0
6.3	3.7
6.4	2.8
7.2	3.0
7.5	2.8
6.8	3.0
7.0	3.2
7.4	3.2
7.0	3.0
6.5	2.5
9.0	4.5
7.2	3.0
6.7	2.4
7.0	2.9
7.1	3.8
7.4	3.3
6.2	2.5
7.0	3.0
7.4	3.0
6.3	2.5
6.5	2.8
11.0	4.3
6.2	2.5
7.1	3.0
6.4	3.0
7.5	3.0
7.9	3.3
7.2	3.5
6.6	3.2
